# Quantification of network structural dissimilarities

**DOI:** 10.1038/ncomms13928

**Published:** 2017-01-09

**Authors:** Tiago A. Schieber, Laura Carpi, Albert Díaz-Guilera, Panos M. Pardalos, Cristina Masoller, Martín G. Ravetti

**Affiliations:** 1 Departmento de Engenharia de Produção, Engineering School, Universidade Federal de Minas Gerais, Avenida Antonio Carlos 6627, Belo Horizonte 31.270-901, Brazil; 2 Departament de Física, Universitat Politècnica de Catalunya, 08222 Terrassa, Spain; 3 Departament de Física Fonamental, Universitat de Barcelona, 08028 Barcelona, Spain; 4 Universitat de Barcelona, Institute of Complex Systems (UBICS), 08028 Barcelona, Spain; 5 Industrial and Systems Engineering, University of Florida, Gainesville, Florida 32611-6595, USA

## Abstract

Identifying and quantifying dissimilarities among graphs is a fundamental and challenging problem of practical importance in many fields of science. Current methods of network comparison are limited to extract only partial information or are computationally very demanding. Here we propose an efficient and precise measure for network comparison, which is based on quantifying differences among distance probability distributions extracted from the networks. Extensive experiments on synthetic and real-world networks show that this measure returns non-zero values only when the graphs are non-isomorphic. Most importantly, the measure proposed here can identify and quantify structural topological differences that have a practical impact on the information flow through the network, such as the presence or absence of critical links that connect or disconnect connected components.

## Abstract (web summary)

Identifying and quantifying dissimilarities among graphs is a problem of practical importance, but current approaches are either limited or computationally demanding. Here, the authors propose an efficiently computable measure for network comparison that can identify structural topological differences.

Quantifying dissimilarities and determining isomorphisms among graphs are fundamental open problems in computer science, with a very long history^1^,^2^,^3^,^4^,^5^,^6^,^7^,^8^,^9^,^10^,^11^,^12^,^13^,^14^,^15^. The graph isomorphism problem consists in deciding whether two graphs are identical, presenting a one-to-one correspondence between its components. This problem holds a special place in the complexity theory field, as no polynomial time algorithm is still known. Thus, its complexity remains undefined since the mid-70s. A recent work proposed a quasi-polynomial time algorithm^16^, which checks subsections of the graphs for isomorphism, through a series of simple means. However, the problem remains, for highly symmetric structures that are still very expensive to compute^17^,^18^.

In practice, the quantification of graph dissimilarities brings much more information about the graphs than the binary answer to the graph isomorphism problem. Similarity measures have many uses due to the current widespread use of networks in social sciences, medicine, biology, physics and so on^19^,^20^,^21^,^22^,^23^,^24^,^25^,^26^,^27^,^28^,^29^,^30^. They can help, among many other examples, to discriminate between neurological disorders by quantifying functional and topological similarities^31^, to find structurally more similar molecules that are more likely to exhibit similar properties, for drug design^32^, and to quantify changes in temporal evolving networks^22^.

Most methods for graph comparison have shown to be efficient for specific purposes, but the information they provide is often limited or incomplete. Important structural differences are missed or underestimated, because the measure employed considers graph properties that only partially describe the graphs^33^.

Regarding network functionality, it is important that a dissimilarity measure captures and adequately quantifies topological differences. A good dissimilarity measure should have the ability to recognize the different roles of links and nodes, considering disconnections and other structural conditions.

The goal of this work is to propose a discriminative and computationally efficient metric to distinguish and quantify graph dissimilarities. We define a dissimilarity metric able to identify and quantify topological differences. The main idea to measure the dissimilarity, *D*(*G*, *G*′), of two graphs containing directed or undirected links is to associate to each structure a set of probability distribution functions (PDFs), representing all node's connectivity distances, and compare them, by standard information-theoric metrics. We consider three distance-based PDF vectors in a three-term function. The first term compares networks, through their network's distance distributions, capturing global topological differences. The second term compares the connectivity of each node and how each element is connected throughout the network, by looking at the node's distances distributions. The last term analyses the differences in the way this connectivity occurs, through the analysis of the alpha centrality.

The *D*-measure (*D*) allows one to compare networks efficiently and with high precision. We prove that isomorphic graphs present a zero distance. Extensive computational experiments show that, *D*, do not present any counterexample when recognizing non-isomorphic structures. We also find that the measure is able to characterize the evolution of dynamical systems, being able to identify the small-world region in the Watts–Strogatz process (WS) and phase transitions in Erdös–Rényi (ER) network's evolution. Considering real networks, *D* evaluates the goodness of the adjustment of network models and predicts their critical percolation probabilities.

## Results

### *D*-measure

**Figure 1:**
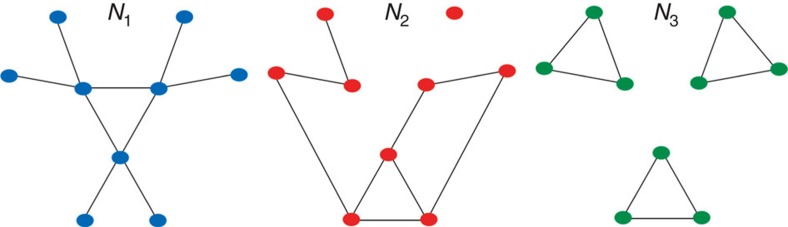
Introductory example. Schematic representation of three different networks with the same number of nodes and links. Table 1 depicts Hamming distance, GED and the proposed dissimilarity measure between the networks.

We introduce *D* with a simple example. Figure 1 displays three networks with nine nodes and nine links, representing different topologies: *N*1 has no disconnections, *N*2 has one disconnected node and *N*3 is disconnected into three connected components. Table 1 depicts results for two popular distance measures, Hamming (H)^34^ and graph edit distance (GED)^35^. As it can be seen in this example, they do not capture relevant topological differences, returning the same distance value for all comparisons and missing the fact that *N*3 is totally disconnected.

A good measure should return a higher distance value between *N*1 and *N*3, than between *N*1 and *N*2. Differently of *N*2, that has only one disconnected node, *N*3 presents three connected components, completely interrupting the information flow through the network. Interesting comparisons are also pairs *N*1–*N*3 and *N*2–*N*3. The measure should recognize *N*3 as more similar to *N*2 than to *N*1, as both *N*3 and *N*2 have disconnected elements.

We begin by defining the concept of network node dispersion (NND). The NND is a measure of the heterogeneity of a graph *G* in terms of connectivity distances. We qualify a network as heterogeneous when it possesses a high diversity of node-distance patterns and, consequently, a high NND value. NND will be used in the definition of *D*(*G*, *G*′). It is computed by the Jensen–Shannon divergence, a dissimilarity measure among *N* PDFs^36^.

To perform a highly precise comparison, instead of using vectors in which the elements are numbers (for example, the number of links of each node), we consider vectors in which the elements are PDFs; specifically, the distance distribution in each node *i*, **P**_i_={*p*_i_(*j*)}, with *p*_i_(*j*) being the fraction of nodes that are connected to node *i* at distance *j*. The set of *N* node-distance distributions, {**P**_1_ … **P**_N_}, contains detailed information of the topology of the network, in a compact way. From this set, the network's degree distribution, the network's distance distribution and several other features can be deduced (see Supplementary Note 1).

Considering a network with *N* nodes, the set of *N* distance distributions {**P**_1_ … **P**_N_}, is normalized by log(*d*+1), being *d* the network's diameter. Then, NND is defined as:


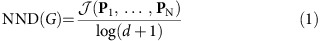


with 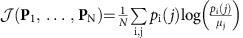 and 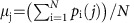 being the Jensen–Shannon divergence and the average of the *N* distributions, respectively.

We illustrate the properties of NND with two numerical experiments, using well-known network models.

**Figure 2:**
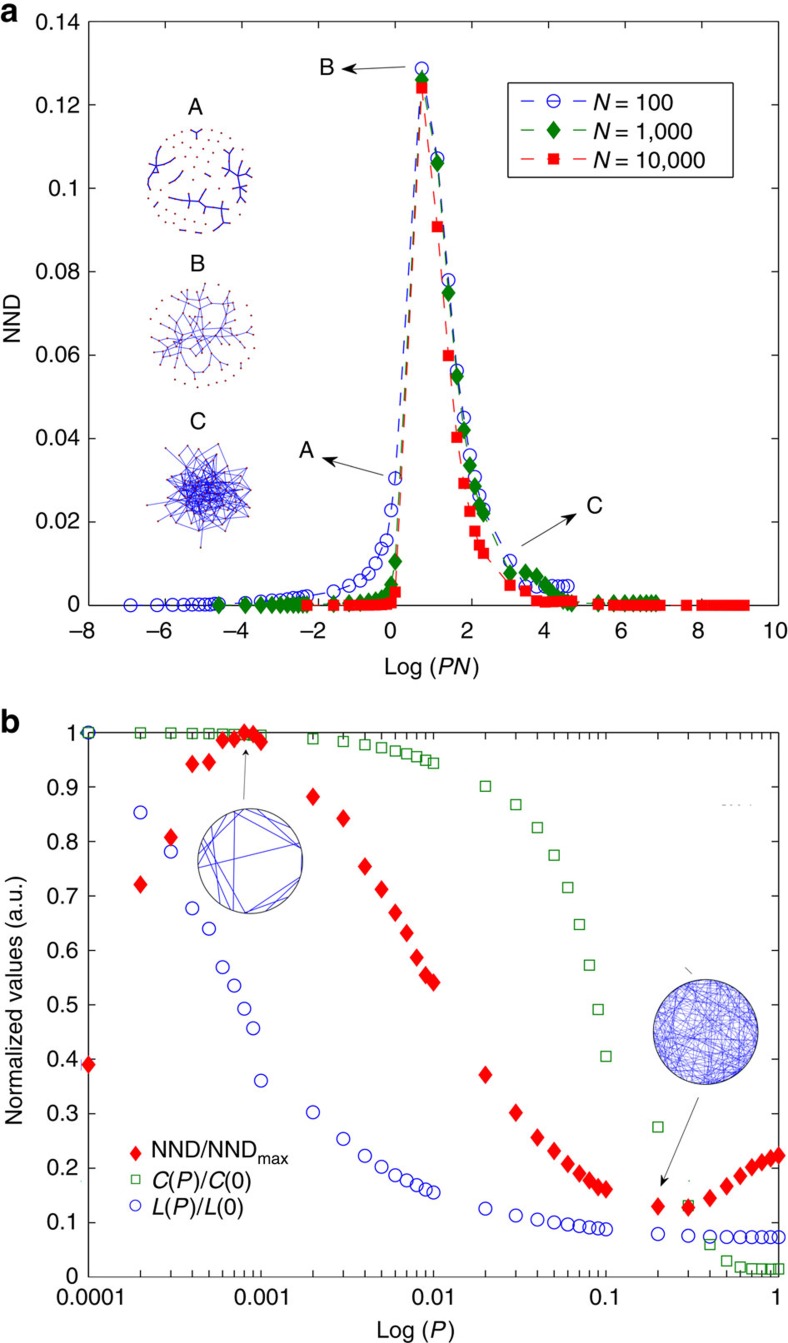
Network node dispersion. (**a**) Average results for 100 independent ER networks of sizes *N*=10^2^, 10^3^ and 10^4^ versus the connection probability *P* (logarithmic scale). (**b**) Average normalized NND, path length (*L*) and clustering coefficient (*C*) for 100 independent WS networks (*N*=10^3^ and average degree 10) versus the rewiring probability *P* (logarithmic scale). The highlighted networks illustrate the topology for the maximum and minimum NND value.

The first one considers 100 ER networks^37^ generated by randomly connecting pairs of nodes with probability *P*. Different network sizes (*N*=10^2^, 10^3^ and 10^4^) and different probability values are considered. At low *P* values the network consists of a set of small connected components and when increasing *P* above a critical value, *P*_c_=1/*N*, the network collapses in a single large connected component, corresponding to the percolation transition. Figure 2a depicts how the NND detects this transition for all sizes considered, being *P*_c_ the last point before the peak. We also note that the maximum NND value (*P*≈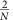) possesses a very low variation as *N* increases (Supplementary Fig. 1).

The second experiment consists of 100 realizations of the WS rewiring model^38^. The number of nodes (*N*=10^3^) and number of links are constant, corresponding to an average degree equal to 10. Figure 2b shows NND versus the rewiring probability, *P*, in logarithmic scale. We observe that the NND allows delimiting the small-world region between its maximum and minimum values: maximum NND indicates maximum connectivity heterogeneity, whereas minimum NND indicates that the nodes are more homogeneously connected.

As shown by the previous examples, NND captures relevant features of a network and thus it can be used for network comparison. However, most *k*-regular networks (graphs in which all nodes have degree *k*) possess NND=0. To define a general dissimilarity measure, it is important to properly discriminate them.

To take this into account, we also consider for the definition of the dissimilarity measure, the difference between the graphs averaged node-distance distributions (network's distance distribution), *μ*_G_ and *μ*_G′_, and the comparison between the *α*-centrality values of the graphs and their complements^39^, computed through the Jensen–Shannon divergence (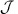) (see Supplementary Note 2).

Then, the dissimilarity measure proposed is


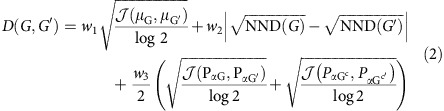


where *N* and *M* are the sizes of *G* and *G*′, respectively, and *G*^*c*^ indicates the complement of *G*. As the NND is always <1 and 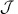
(*P*_G_, *P*_G′_)/log 2≤1 then, 0≤*D*(*G*, *G*′)<1. *w*_1_, *w*_2_ and *w*_3_ are arbitrary weights of the terms where *w*_1_+*w*_2_+*w*_3_=1; however, after extensive experimentation we selected the following weights *w*_1_=*w*_2_=0.45 and *w*_3_=0.1 as the most appropriate to quantify structural dissimilarities in networks. Supplementary Note 3 shows that the choice of the weights does not change the metric character and presents a discussion regarding the weights selection. This approach can be easily adapted to compare networks of different number of nodes, as discussed in Supplementary Note 4.

Defined in this way, *D* captures global and local graphs dissimilarities. The first term compares averaged connectivity node's patterns, corresponding to the so-called graph distance distribution^28^. Graphs sharing the same distance distribution present the same diameter, average path length (APL) and other connectivity features.

The second term analyses the heterogeneity of the nodes. Graphs presenting the same NND are graphs that have the same connectivity distance profile.

The third term considers the centrality of each node, taken into account each node's direct and indirect connectivity span. When considering the graph's complement, the measure also captures the effect of disconnected nodes. This term is the only one able to discriminate between complete graphs of different sizes and also among other distance-regular structures such as the Desargues and dodecahedral graphs (see Supplementary Fig. 2a).

*D*(*G*, *G*′) identifies and properly quantifies structural topological differences, which affect the information flow through the networks. This can be seen in Fig. 1 and Table 1, in which increasing topological differences correspond to higher *D*-values.

### Isomorphism

By performing extensive experiments in synthetic and real-world networks, we show that *D*(*G*, *G*′) recognizes isomorphic graphs, returning non-zero values when the graphs are non-isomorphic.

We note that *D*(*G*, *G*′)=0 only if *G* and *G*′ have the same graphs distance distribution, the same NND and the same *α*-centrality vector. However, there is no guarantee that *D* returns a non-zero value for all non-isomorphic networks. In other words, it is possible to obtain *D*(*G*, *G*′)=0 even if *G* and *G*′ are not isomorphic. To investigate this limitation, we analysed all non-isomorphic graphs of size 6, 7, 8 and 9. For graphs with 20 nodes, we focused on the worst cases for *D*, *k*-regular connected graphs with degrees varying from 2 to 11. Finally, we also generate all non-isomorphic trees with 20 and 21 nodes. After ∼10^12^ comparisons, results demonstrate the high accuracy of the proposed measure for recognizing the non-isomorphic condition, without any counter-example (see Data availability in Methods for instances and algorithms).

Most importantly, we observe that, from a computational perspective, the time complexity of the algorithm is polynomial, as it relies on the computation of all shortest paths length, that is known to be a polynomial problem^40^, that by using Fibonacci heaps can be implemented in *O*(*E*+*N* log*N*)^41^. The Hamming distance is computed in polynomial time, only when nodes are labelled, as it consists in a matrix difference *O*(*N*^2^). However, the problem with H is the lack of information, as it only considers the number of missing links and not their role in the topology structure. In the case of GED, its computation corresponds to a NP-Hard problem^2^, being very unlikely to expect a polynomial approach to compute it. Besides the major drawback of an exponential computational time, the usefulness of its results as a measure of dissimilarity is at least questionable. As it can be seen in Fig. 1 and Table 1, neither H or GED can properly detect and manage network disconnections. Supplementary Note 5 and Supplementary Tables 1 and 2 present algorithms found in the literature, either to solve the isomorphism problem or to compute a dissimilarity measure between networks; this compilation briefly describes their main characteristics, drawbacks and results.

### Classical models

We consider five networks with 20 nodes and 40 links: a four-regular network (R), a random network (ER), two small-world structures with *P*-values corresponding to the lowest and highest NND (WSMIN and WSMAX), and a scale-free Barabási–Albert (BA) network with parameter *m*=2 (ref. 42).

The lowest *D*-value is obtained between ER and WSMIN. This is expected due to the fact that the WS process transforms a *k*-regular lattice into a random structure by rewiring links. *D* recognizes the small difference between them, as the intrinsic memory of the WS process does not allow the network to evolve to a pure ER structure^43^. However, when these two structures are compared against other networks, the differences captured by *D* show no statistical significance. See Supplementary Table 3 for values and confidence intervals.

**Figure 3:**
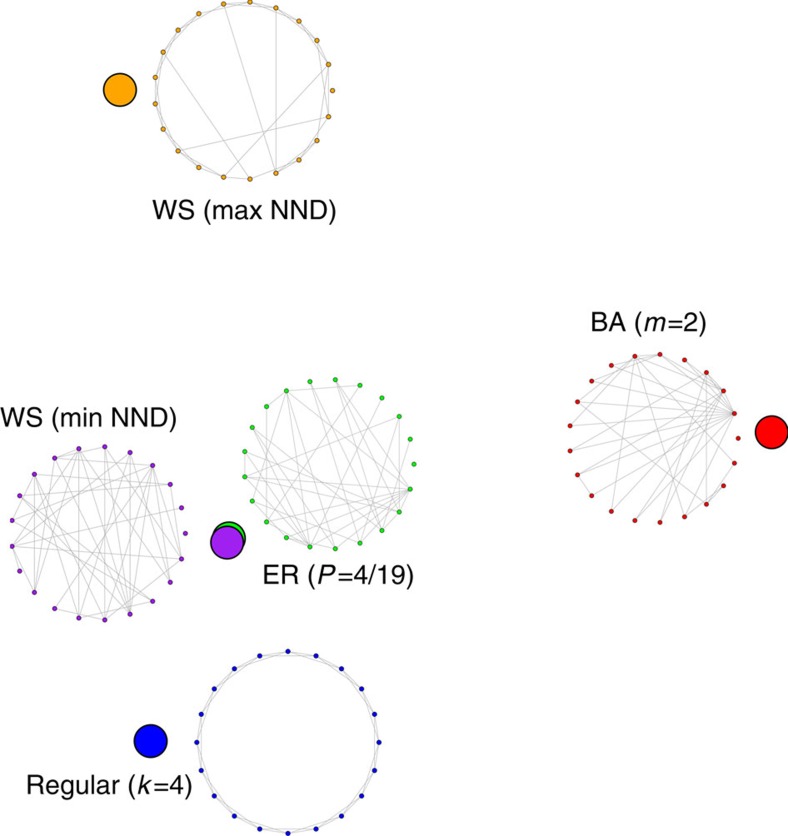
Two-dimensional scaling map for classical models. Schematic representation of five topologically different networks through a multidimensional scaling (MS) map of the *D* average values between all pairs of networks in Supplementary Table 3. In MS, the cartesian coordinates are chosen so the 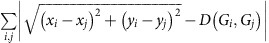 is minimized.

In contrast, the highest *D*-value is obtained for BA and WSMAX, followed by BA and R. The BA network corresponds to the most complex structure from the five here studied. In terms of node distances distributions, the BA structure possesses low node-distance heterogeneity, as a great number of nodes are connected to hubs, in a similar way. Thus, *D* considers BA closer to R than WSMAX. WSMAX corresponds to a stage in the WS process in which the number of shortcuts created in the network generates a decrease in the APL, increasing the node-distance heterogeneity. Besides the low values of APL, BA structures are known to present low clustering coefficient, features also present in ER and WSMIN. *D* acknowledges this fact by locating them closer to BA. Figure 3 depicts a schematic representation of the networks obtained through a multidimensional scaling map of the *D*-values between all pairs of networks presented by increasing averaged values over 1,000 experiments.

**Figure 4:**
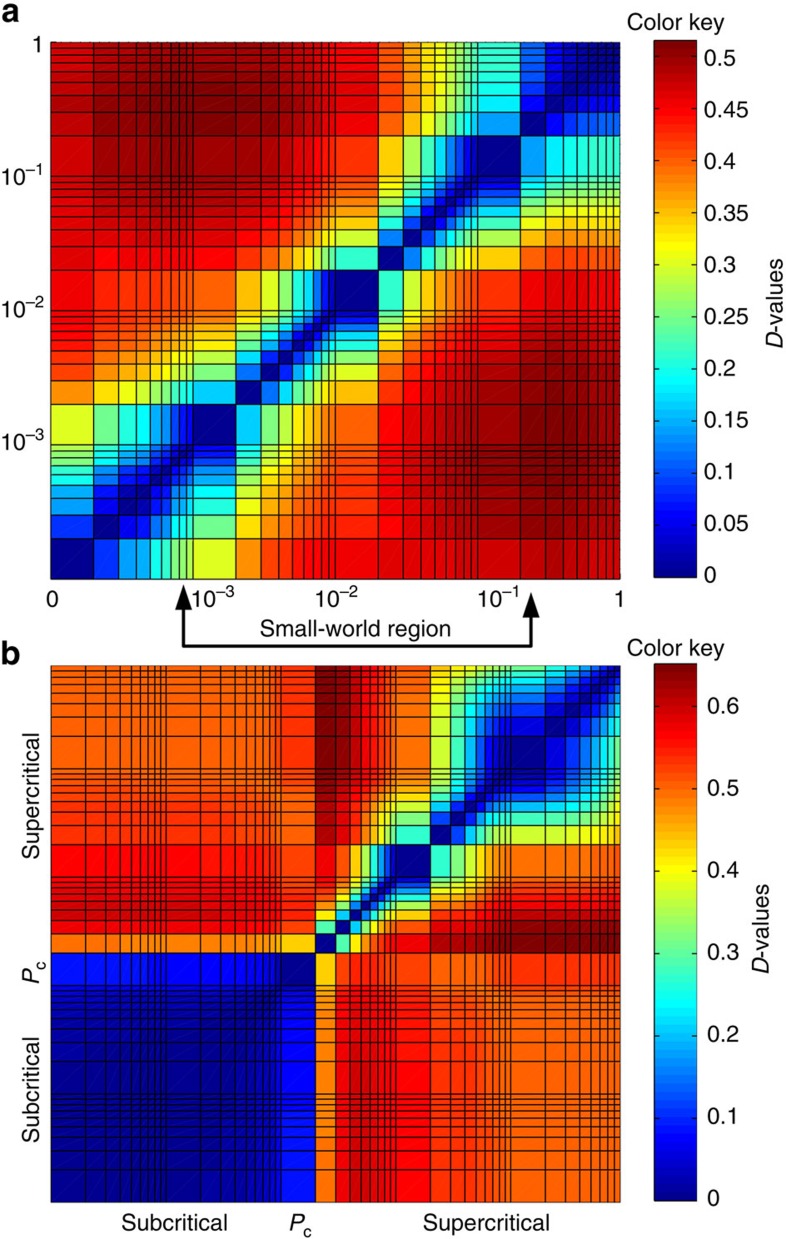
*D*-measure for classical evolving models. (**a**) Dissimilarity values for each pair of networks created in the WS rewiring of size *N*=10^3^, average degree 10 and different values of the rewiring probability *P* (see Fig. 2b). The axes are in logarithmic scale. In the heatmap are depicted the largest dissimilarity values without considering the *k*-regular lattice, arrows mark their position and the small-world region between them. (**b**) Dissimilarity values for each pair of networks in the ER process (see Fig. 2a). We consider size *N*=10^3^, for different values of connection probability *P*.

For the following example, we first consider synthetic networks generated by WS and ER processes. Figure 4a depicts the dissimilarity value for all pairs of networks of size *N*=10^3^ constructed during the WS process. The first row and column represent the distance between all graphs and the initial lattice. The maximum dissimilarity value, not considering comparisons with the initial lattice, coincide with the maximum and minimum NND values, delimiting the small-world region. It can be seen that networks corresponding approximately to *P*<10^−3^ are very similar between each other and they become gradually more dissimilar to networks generated with higher *P*-values. For networks in the region 2 × 10^−1^<*P*<1, they are similar to each other, but very dissimilar to initial networks. Finally, networks corresponding to probabilities in the interval 10^−3^<*P*<2 × 10^−1^ are dissimilar to networks of both extremes of the process, delimiting the small-world region.

Figure 4b shows the dissimilarity values *D* for all pairs of ER networks of size *N*=10^3^. *D* clearly captures the topological phase transition at *P*_c_. As expected, higher values are obtained when comparing networks with *P* below and above the critical value. We also note that networks with *P*<*P*_c_ are more similar among each other than networks with *P*>*P*_c_.

### Percolation on real networks

**Figure 5:**
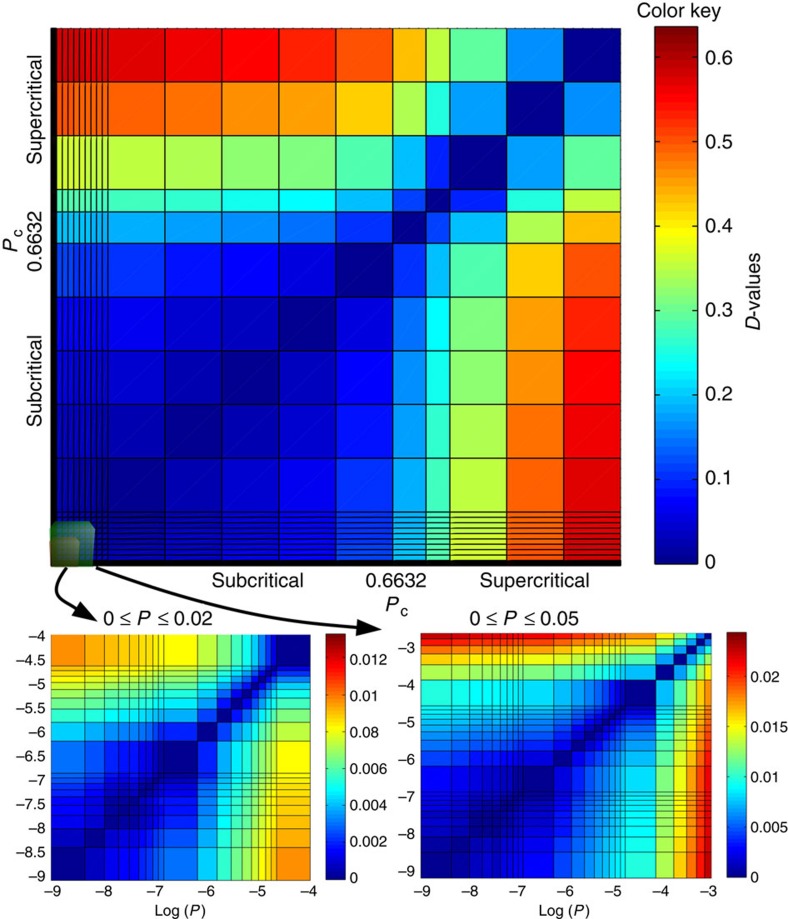
Percolation on the Power Grid network. Heatmap of the dissimilarity function highlighting the regime of large percolation thresholds for the Power Grid network. This network also contains a double phase transition characterized by lower(s) thresholds that does not coincide with the largest percolation strength. For small values of *P*, the dissimilarity function show two small percolation phase transitions (see Supplementary Note 6 and Supplementary Fig. 7 for more information).

The phase transition captured by the dissimilarity function in the ER model represents the bond percolation threshold on complete graphs; however, as this measure captures abrupt changes in distances within the network, it also captures the existence of a percolation threshold in real networks. Figure 5 shows how *D* captures the largest percolation transition in the Power Grid network (*P*_c_≈0.6632) and also a double phase transition characterized by two small peaks in the susceptibility function at *P*≈8 × 10^−5^ and *P*≈6 × 10^−3^, as depicted in the two small figures.

We propose here an algorithm based on the hypothesis that, when looking for the phase transition, two networks in the subcritical or supercritical phases present smaller *D*-values than a pair of graphs with one in each phase. By applying a bisection method-like procedure, we obtain good approximations of the percolation transition with a low number of simulations. We compare our results against the Monte Carlo (MC) algorithm proposed by Newman and Ziff^44^. We follow the instructions used by Radicchi^45^, where an extensive empirical experiment was performed using MC.

The algorithm begins with two probabilities, *β* and *α*, respectively, on the supercritical and subcritical phases. We compute the mean value of these probabilities *P*_m_=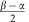 and through a series of simulations we estimate the distance between their correspondent averaged graph structures. If *D*(*G*_m_, *G*_*α*_)>*D*(*G*_m_, *G*_*β*_) then *β*=*P*_m_ else *α*=*P*_m_, when the distance between *β* and *α* reaches a precision value (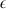), the algorithm stops returning 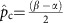. Table 2 depicts results for a set of real networks. Supplementary Note 6 presents a pseudo-code and a detailed explanation of the experiment.

In terms of computational complexity, after the first iteration, our algorithm computes *s* different networks per iteration and each corresponding NND. Thus, per iteration, our algorithm has a complexity 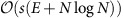, considering 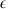
=0.01, *α*=0 and *β*=1, we need to perform seven iterations. For the specific example of the Power Grid network, with *s*=100, our algorithm needs ∼5,500 s, against the 35,000 s of the MC with 10,000 iterations (CPU times of both algorithms can be improved with a good *P*_c_ approximation value). By increasing *s* and reducing 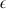, we can improve the algorithm precision, which can also be used as a warm start for the MC procedure.

### Model selection

We consider here the problem of choosing the most appropriated model to simulate real systems. In this experiment, we use *D* to compare real networks with well-known null models including, Molloy–Reed (MR)^46^, Maslov–Sneppen (MS)^47^ and dk model^25^. MR is a null model that preserves the degree distribution of the network, but the connection structure is lost. MS is a null model where links are randomly rewired. Its default setting considers 4|*E*| rewiring procedures. However, exists an appropriate number of rewiring operations from which MS can be considered equivalent to MR. Finally, we consider the dk models for different *k*-values (1.0, 2.0, 2.1 and 2.5). *k*=1.0 generates networks preserving the degree sequence and, as it can be seen in Supplementary Note 7, it is equivalent to MR and MS null models. *k*=2.0 preserves the degree sequence and degree correlation; *k*=2.1 also preserves the clustering coefficient and finally *k*=2.5 includes the clustering spectrum.

**Figure 6:**
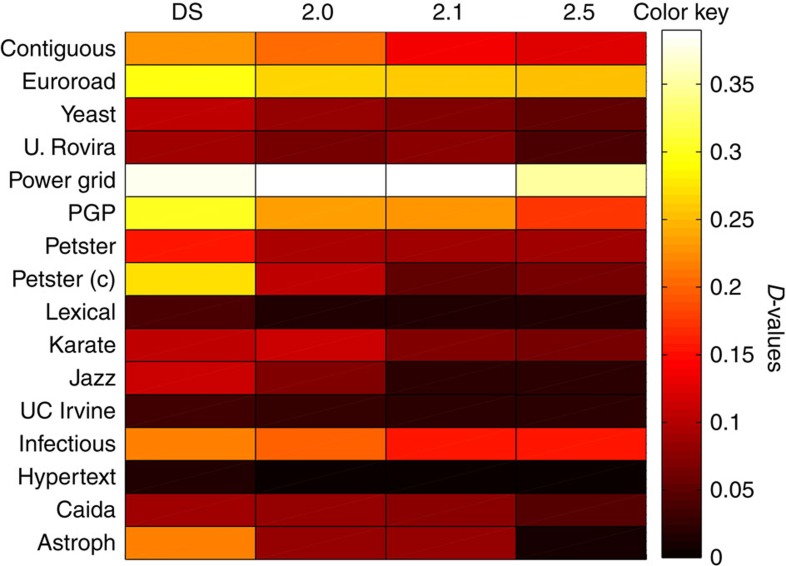
dk Models. Dissimilarity values between real-world networks and four null models. From left to right we report averaged results after 30 independent runs; DS, degree sequence (MS, MR and *k*=1.0), generates equivalent results and the last three columns are obtained using the dk model for different *k*-values (2.0, 2.1 and 2.5).

Each model is run 30 independent times and the averaged *D*-values are presented in Fig. 6. When preserving only the degree sequence, the null models capture some topological features; however, they have no information regarding node's correlation and global connectivity patterns. It can be seen from Fig. 6 that, as expected, *D* decreases as parameter *k* increases.

It is worth noting that, in most cases, transitions from *k*=1.0 to *k*=2.0 and from *k*=2.0 to *k*=2.1 present significative differences (see confidence intervals in Supplementary Table 4). That is not always the case for transitions between *k*=2.1 to *k*=2.5. Results for the Petster (C) network show that models considering *k*=2.1 are closer to the real network than models *k*=2.5; this can be the case of an outlier network as discussed in ref. 25. After the analysis of the generated networks, we could verify that *k*=2.1 produce networks with closer APL (3.558) than their *k*=2.5 counterpart (3.502) and both overestimate the network diameter 16.21 and 15.6; these are average results over 30 runs. The original network has APL=3.588 and diameter 10.

It is interesting noticing that the Power Grid and Euroroad networks show significative higher distances to the dk model when compared with all other real networks. This poor adjustment of the dk model to the Power Grid network is also discussed in refs 25, 48.

### Distances between real networks

We use the dissimilarity measure *D* to compare real-world networks. We consider 16 data sets of 9 network types: computer, online contact, communication, human contact, infrastructure, lexical, metabolic, social and co-authorship. All networks are freely available at The Koblenz Network Collection^49^ (see description in Supplementary Note 8).

**Figure 7:**
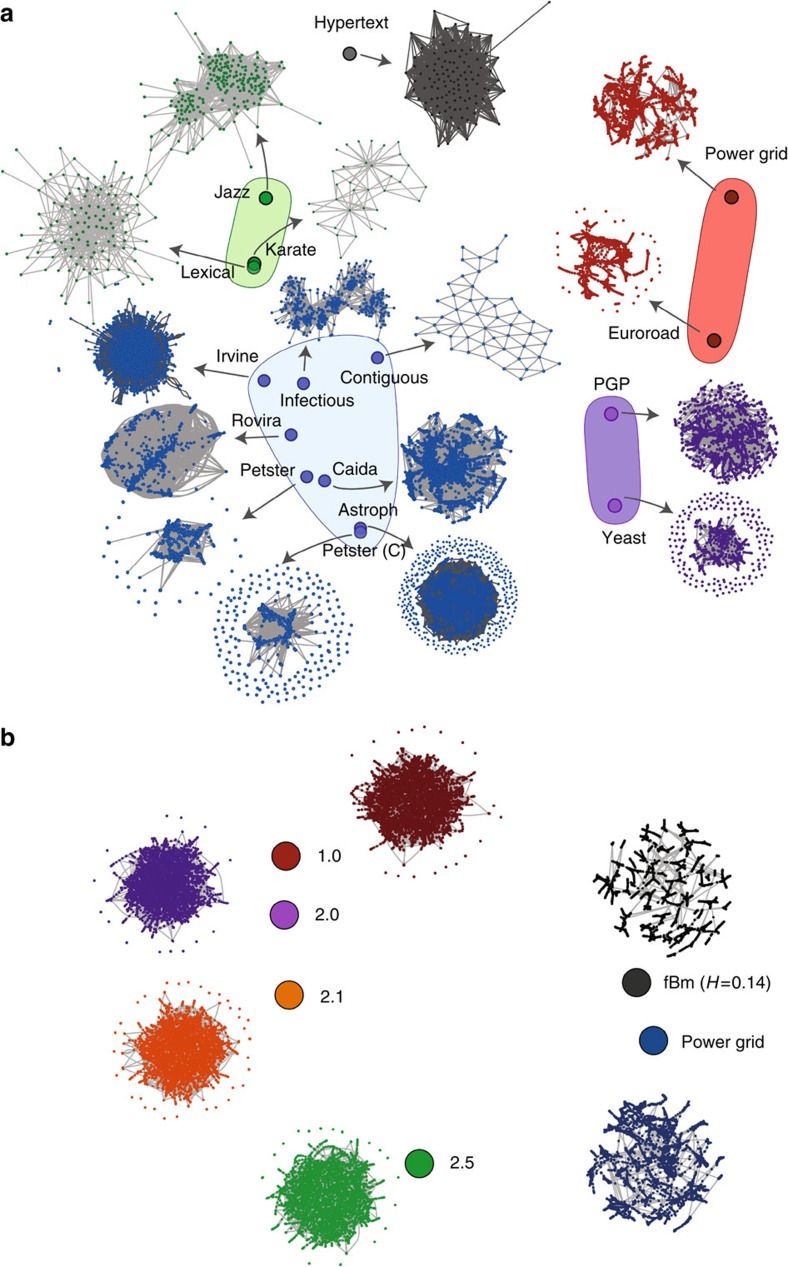
Multidimensional scaling maps for real-world networks. (**a**) Multidimensional scaling map of the set of real networks performed over the averaged *D*-values. (**b**) Multidimensional scaling map of the Power Grid network, and the best *D* approximation for the dk model and a fBm-derived network. The fBm (*H*=0.14) network through horizontal visibility graph (HVG) is closer to the Power Grid network, without using any information from the network.

Figure 7a depicts *D*-values between all pairs of networks. Remarkably, Social Networks appear to be very similar to each other, in good agreement with previous observations^50^. In addition, we can observe that CAIDA, a computer type network, is similar to communication, social, co-authorship and the human contact infections socio-patterns network. The infrastructure networks (Power Grid and Euroroad) are the most different with respect to the entire group, but similar to each other. Both networks present particular characteristics, as scarcity due to physical constraints, presenting neither a scale-free nor a classical small-world behaviour^51^,^52^. A tree-like structure, which is also possible to visualize in Fig. 7a, is a common feature in these networks. *D* captures this structural pattern differentiating them from all other topologies.

We compare these networks (Power Grid and Euroroad) with other well-known tree-like structures, as are the case of networks constructed via the horizontal visibility graph^53^, from fractional Brownian motion (fBm) time series, with different Hurst exponents (*H*)^54^. We found that these networks posses significantly lower distances to fBm networks than to the dk model. This can be seen in Fig. 7b, in which we compare distances between the Power Grid network with networks generated by dk model and also with an fBm (H=0.14) network (see Supplementary Note 9).

### Brain networks

As a final application, we perform a study to compare brain networks constructed through electroencephalography exams (EEG). The data contain measurements from 64 electrodes placed on the subject's scalps sampled at 256 Hz (3.9 ms epoch) during 1 s^55^. The full data set contains 120 trials for 122 subjects; however, as some samples are incomplete, we consider only the 107 subjects with complete trials (39 control and 68 alcoholic samples).

For each subject, a weighted network of the entire brain is created following the method described in ref. 56. However, instead of using a linear correlation measure between the time series, we transform them into a graph via horizontal visibility graph algorithm^53^ and we consider the correlation between each pair of regions as given by 1 minus the dissimilarity *D* (1−*D*(*G*, *G*′)). The resulting network represents the weighted similarity between brain regions, allowing comparisons between individual brain networks.

**Figure 8:**
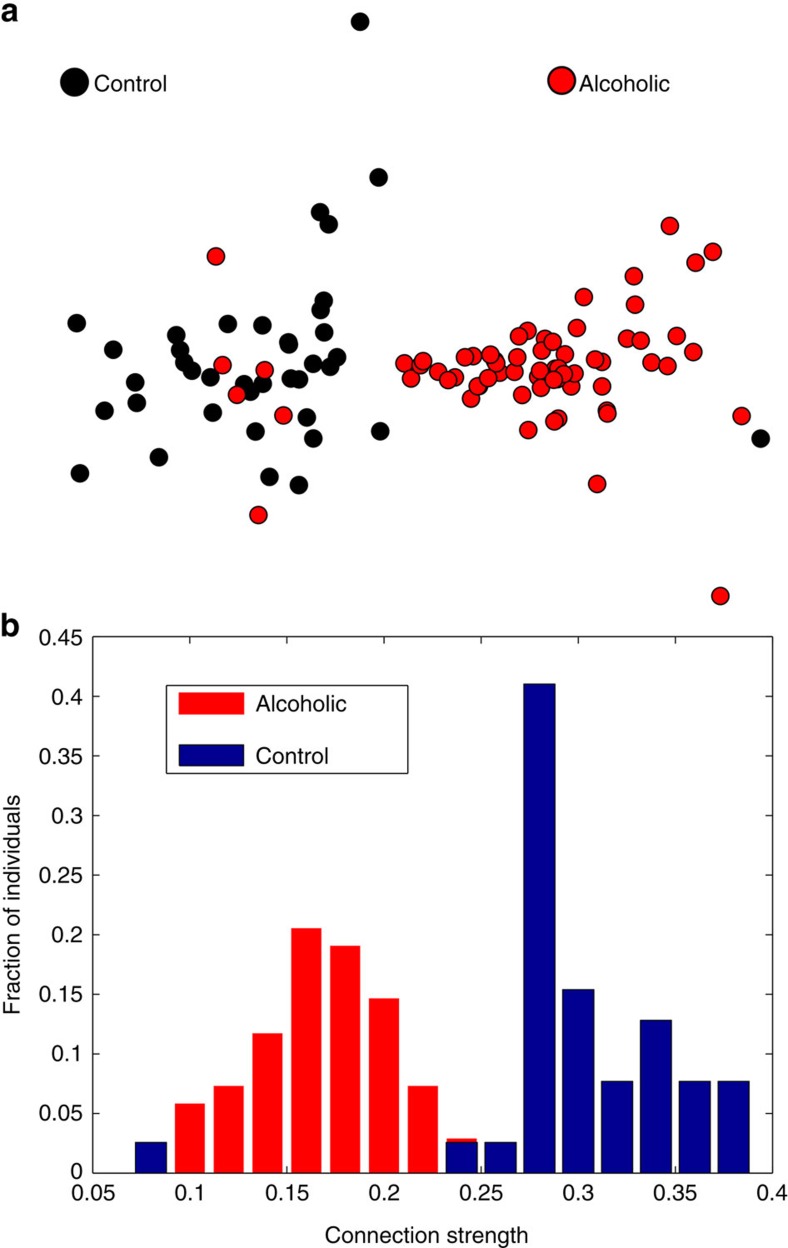
Brain network application. (**a**) Multidimensional scaling map of the values between all pairs of brain networks presented by differences among connection strengths between regions ‘Y' and ‘nd'. (**b**) Histogram of the connection strengths between regions ‘Y' and ‘nd'.

By using this straightforward methodology, we are able to detect two regions of the brain called ‘nd' and ‘y', where the weight of the connections between these regions is higher in control than in alcoholic networks, as shown in Fig. 8. Supplementary Fig. 9 depicts the results of applying the same methodology but considering the Hamming distance, in which it is possible to see that it is not capable of distinguishing between the groups.

## Discussion

*D* is a highly precise network dissimilarity measure, based on three distance-based PDF vectors extracted from the graphs and defined as a three-term function. It compares, through the Jensen–Shannon divergence, topological differences between networks. Through extensive numerical experiments, we show that *D* appropriately captures topological differences between networks and returns *D*=0, when comparing isomorphic graphs. Non-zero *D*-values indicate a non-isomorphic condition and represent a quantification of the topological difference between them.

*D* is able to identify the small-world region in a WS process and phase transitions in ER network's evolution. Considering real systems, *D* evaluates the goodness of the adjustment of network models and predicts their critical percolation probabilities.

One aspect we must point out is that the use of *D* to compare sparse graphs, as it is the case of real-world networks, implies in processing dense graphs when computing the *α*-centrality of their graph's complements, increasing the computational cost. However, as the use of the third term (*α*-centrality) is only strictly necessary to distinguish highly regular structures, *D* can be computed avoiding the third term of the equation, without significant precision loss.

*D* also have many practical uses, that among many others, we can mention applications in image and pattern recognition and in the characterization of time-evolving networks. *D* can be employed in the design of accurate classifiers for biological networks and is a promising tool to study different aspects of multilayer networks.

### Data availability

All relevant data and algorithms are publicly available at https://github.com/tischieber/Quantifying-Network-Structural-Dissimilarities.

## Additional information

**How to cite this article:** Schieber, T. A. *et al*. Quantification of network structural dissimilarities. *Nat. Commun.* **8,** 13928 doi: 10.1038/ncomms13928 (2017).

**Publisher's note**: Springer Nature remains neutral with regard to jurisdictional claims in published maps and institutional affiliations.

## Supplementary Material

Supplementary Information Supplementary Figures, Supplementary Notes, Supplementary Tables and Supplementary References.

## Figures and Tables

**Table 1:** Comparisons between dissimilarity distances.

**Networks**	**H**	**GED**	***D***
(*N*1, *N*2)	12	6	0.252
(*N*1, *N*3)	12	6	0.565
(*N*2, *N*3)	12	6	0.473

*D*, dissimilarity; GED, graph edit distance; H, Hamming.

H, GED and *D* measure (equation (2)) computed for the networks presented in Fig. 1.

**Table 2:** Percolation critical values of real networks.

**Network**	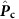	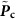	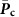
Euroroad	0.6106	0.5823	0.5791
Jazz	0.0397	0.0314	0.0339
Hypertext	0.0245	0.0258	0.0270
Infectious	0.0764	0.0778	0.0791
Karate	0.2404	0.2436	0.2412
Contiguous	0.3513	0.3205	0.3448
Petster	0.0327	0.0273	0.0291
Lexical	0.1239	0.1035	0.1108
Rovira	0.0773	0.0646	0.0652
UC Irvine	0.0289	0.0248	0.026
Power grid	0.6826	0.6583	0.6632
Astrophisics	0.0125	0.0133	0.0127
Caida	0.0294	0.0216	0.0232
PGP	0.0698	0.0671	0.0598
Yeast	0.2010	0.2603	0.2598

Form left to right, we report the name of the network, the prediction value obtained using *D* (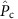) with 100 simulations per distance evaluation, the value obtain by MC after 10,000 simulations (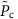) and the MC value after 100,000 simulations (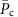).
