# Supplementary material for: Quantification of network structural dissimilarities
Source: Nat Commun. 2017 Jan 9;8:13928. doi: 10.1038/ncomms13928 (PMC5227707; doi:10.1038/ncomms13928)
Supplement: Supplementary Information — Supplementary Figures, Supplementary Notes, Supplementary Tables and Supplementary References. [file ncomms13928-s1.pdf]

SUPPLEMENTARY NOTE 1. PROPERTIES OF THE NODE DISTANCE DISTRIBUTION

The node-distance distribution is a set of probability distributions that associates to each node $i = 1, 2, \ldots, N$ a probability distribution $\mathbf{P}_i(d)$ representing the fraction of nodes connected to i at distance d, where $\mathbf{P}_i(\infty)$ is the fraction of disconnected nodes from i.

The set of node-distance distributions possesses local and global information regarding the connectivity of the network. $(N-1)\mathbf{P}_i(d)$ gives the number of nodes at distance d from i and, thus, the degree (number of nodes at distance 1 from i) and the closeness centrality (the sum of the inverse distance from i to all other nodes) are easily obtained [1]. Regarding global properties, the number and the size of the connected clusters are given by $(N-1)\mathbf{P}_i(\infty)$ which gives the number of nodes in a different cluster from i. The average of the node-distance distributions gives the network distance distribution carrying information of the diameter, average path length, average degree, average cluster size, among others.

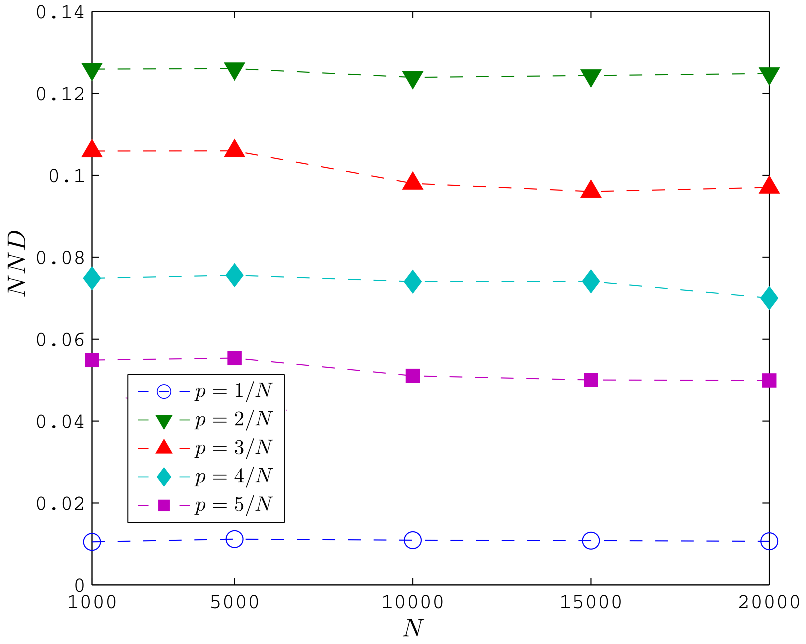

Supplementary Figure 1: **Network Node Dispersion values for Erdös-Rényi networks.** NND values for networks generated by the ER model with different average degree (generation probability).

SUPPLEMENTARY NOTE 2.

Here, we briefly describe the meaning of each term on the right-hand side of Equation (2) in the main text.

The first term compares, via the square root of the Jensen-Shannon divergence the average of the node-distance distributions between the graphs. For each node $i = 1,\ 2, \ldots N$, the node-distance distribution, $\mathbf{P}_i(k)$, is defined as the fraction of nodes at distance k from i. By setting $n_{i,k}$ as the number of nodes at distance k from i, $\mathbf{P}_i(k) = n_{i,k}/(N-1)$. Consequently, the average of the N node-distance distributions, μ, is given by:

$$\mu(k) = \frac{1}{N}\sum_{i=1}^{N}\frac{n_{i,k}}{(N-1)}. \tag{1}$$

By observing that $\sum_{i=1}^{N} n_{i,k}$ gives the double of the number of pairs of nodes at distance k from each other and $N(N-1)/2$ is the total number of possible pairs of nodes, $\mu(k)$ gives the fraction of pairs at distance k from each other. Thus, the first term of the right-hand side of Equation (2) in the main text compares, via the Jensen-Shannon divergence, the distance distribution between the graphs.

The second term compares the NND values between the networks.

The third term deals with differences in the node centrality value computed via the alpha-centrality vector.

Alpha-centrality measures the total number of paths from a node, exponentially attenuated by their length. The alpha-centrality matrix gives the number of paths, attenuated by a parameter $0 \leq \alpha < 1$, between two nodes. It can be written as a power series expansion of the adjacency matrix A, with attenuation parameter α given by:

$$C_\alpha = A + \alpha A^2 + \alpha^2 A^3 + \ldots. \tag{2}$$

It can be proved that this expansion converges for $\alpha < 1/\lambda_{max}$, where λ_{max} is the graph's spectral radius (largest eigenvalue of its adjacency matrix). Considering the Perron-Frobenius theory, in a graph (connected or not), λ_{max} is bounded from above by the maximum degree.

For each node, the alpha centrality is given, considering an exogenous factor vector $\vec{v}$ (here considered as a vector giving the link density of every node) by:

$$c_\alpha(i) = C_\alpha \dot{\vec{v}}^t. \tag{3}$$

Defined in this way, considering $\alpha = 1/N$, the alpha-centrality is bounded from above to 1 (value obtained only for nodes in the full graph). Thus, if V_α is ordered by increasing values of the node alpha-centrality.

$$\mathbf{P}_\alpha = \frac{1}{N}[V_\alpha \ \ N - \sum_{i=1}^{N}(V_\alpha(i))] \tag{4}$$

is a discrete probability distribution associated with the alpha-centrality values. Two networks share the same P_α if they possesses the same sorted alpha-centrality sequences.

The graph complement captures differences in nodes with low centrality values. In particular, nodes with low degree centrality tend to possess a low alpha-centrality value and, its importance may be overlooked. As taking the graph complement, low degree nodes increases in importance highlighting local networks differences.

The role of the alpha centrality can be seen in Supplementary Fig. 2.

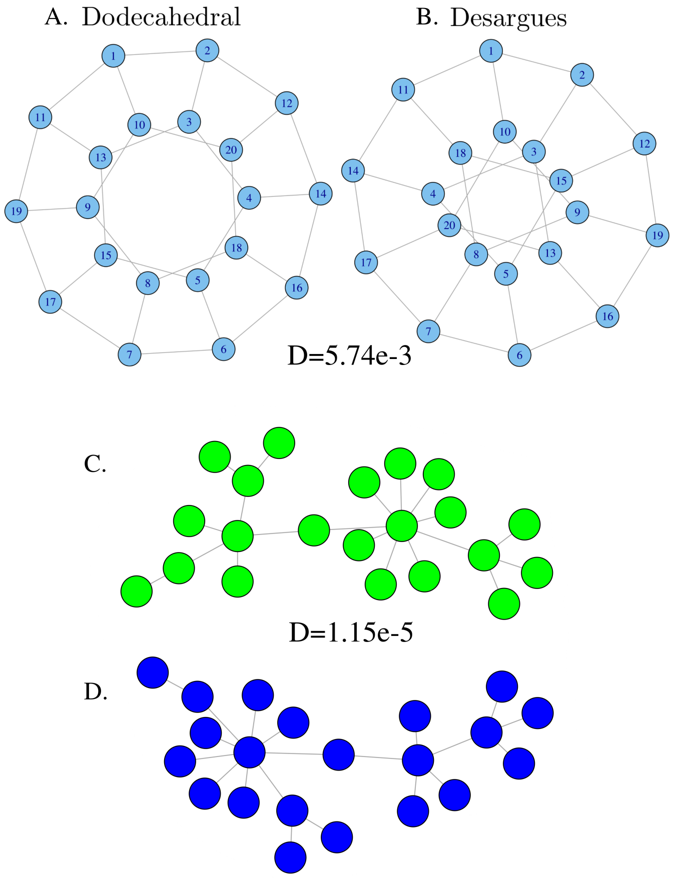

Supplementary Figure 2: **Special cases.** Example of pairs of graphs that have the same NND, and the same distance distributions (dodecaedral (A) and Desargues (B); and two trees of size 21, (C) and (D)). The third term of Equation (2) in the main text, is the only one able to distinguish them. The dissimilarity value is positive for both pairs, confirming their non-isomorphic condition.

SUPPLEMENTARY NOTE 3. PROOF THAT D IS A BOUNDED PSEUDO-METRIC BETWEEN NETWORKS

The dissimilarity $D(G, G')$ between G and G' of size N and M respectively, is as described in the main text, given bys:

$$D(G,G') = w_1\sqrt{\frac{\mathcal{J}_H(\mu_G,\mu_{G'})}{\log 2}} + w_2\left|\sqrt{NND(G)} - \sqrt{NND(G')}\right| + \frac{w_3}{2}\left(\sqrt{\frac{\mathcal{J}_H(P_{\alpha G},P_{\alpha G'})}{\log 2}} + \sqrt{\frac{\mathcal{J}_H(P_{\alpha G^c},P_{\alpha G^{c'}})}{\log 2}}\right) \quad (5)$$

The square root of the Jensen-Shannon divergence is known to be a metric between probability distributions [2]. Thus, $D(G, G') = 0$ means that both networks possesses the same distance distribution, alpha-centrality of the graph and its complement, and NND values. The triangle inequality holds due the fact that $|a + b| \leq |a| + |b|$ for all real numbers a and b, and this result is independent of the chosen weights w_1, w_2, and w_3.

Weight Sensitivity Analysis

To understand the role of the chosen values w_1, w_2 and w_3 we perform a sensitivity analysis. We refer to $D(G, G', w_1, w_2, w_3)$, the dissimilarity between graphs G and G', using weights, w_1, w_2 and w_3.

The first experiment consists of testing link failures on a full graph.

Let G be a full graph with N nodes. After selecting a node, we randomly remove all its adjacent links, and we compute the graphs dissimilarities. We denote G_1, the graph obtained from G, after the removal of a single link, and inductively G_i the graph obtained from G_{i-1} by the deletion of an adjacent link of the selected node.

When considering $N = 100$, the removal of a single edge is better perceived by the third term of the right-hand side of Equation (5). In this case, $D(G_0, G_1, 0, 0, 1) = 0.0502$ is five times the $D(G_0, G_1, 1, 0, 0) = 0.0101$ value and almost twice the $D(G_0, G_1, 0, 1, 0) = 0.0268$ value. Supplementary Fig. 3 - C, shows that the third term is the best in capturing differences in the beginning, and in the end of the link removal process. The behavior exemplifies the role of the third term of the D function, as in these states of the process, the resulting network after a link failure is globally very similar with the original one. The alpha centrality captures the changes caused by the removal of links, in the shortest paths.

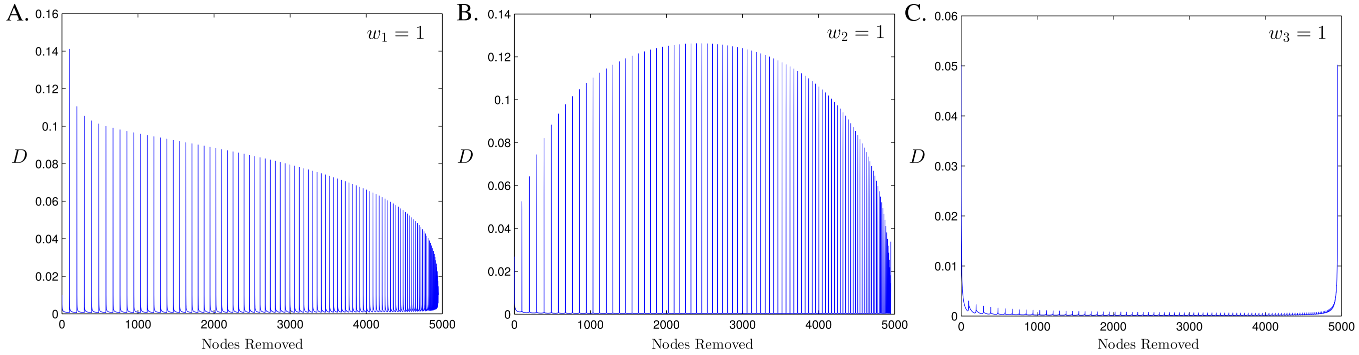

Supplementary Figure 3: **Full graphs attack.** Considering a full graph with 100 nodes, we remove links for a selected node and compute D over consecutive graphs. (A) Measurements considering only the first term of the measure, that is $D(G_i, G_{i-1}, 1, 0, 0)$. (B) Measurements considering only the second term, $D(G_i, G_{i-1}, 0, 1, 0)$. (C) Measurements considering only the third term, $D(G_i, G_{i-1}, 0, 0, 1)$.

Through the deletion of the 99th edge, the network becomes disconnected, causing a big disruption in the distance distribution. Thus, the first term of D, better captures these network changes. $D(G_{98}, G_{99}, 1, 0, 0) = 0.1411$ almost three times the $D(G_{98}, G_{99}, 0, 1, 0)$ and 47 times $D(G_{98}, G_{99}, 0, 0, 1)$ value, detecting the disconnection (Supplementary Fig. 3 - A).

The second term captures the heterogeneity of the topology. The selection of a second node and the removal of the 100th edge, induce high network heterogeneity, presenting a disconnected node, and the connected component

is no longer a full graph. $D(G_{99}, G_{100}, 0, 1, 0) = 0.0489$ almost five times the $D(G_{99}, G_{100}, 1, 0, 0)$ and 21 times the $D(G_{99}, G_{100}, 0, 0, 1)$ value (Supplementary Fig. 3 - B).

As a second experiment, we repeat the percolation tests in the Erdös-Rényi process, using only the alpha centrality term. Supplementary Fig. 4 shows that the alpha centrality, $D(G, G', 0, 0, 1)$, is not capable of detecting the phase transition captured when using D, $D(G, G', 0.45, 0.45, 0.1)$, (see Fig. 4-B in the main text). The maximum NND value for this case is 0.12. A high value of w_3 would distort the D values, minimizing the power of the NND in detecting these important topological changes. Considering this, we fixed $w_3 = 0.1$, value that only significantly alters D when comparing highly distance-regular graphs.

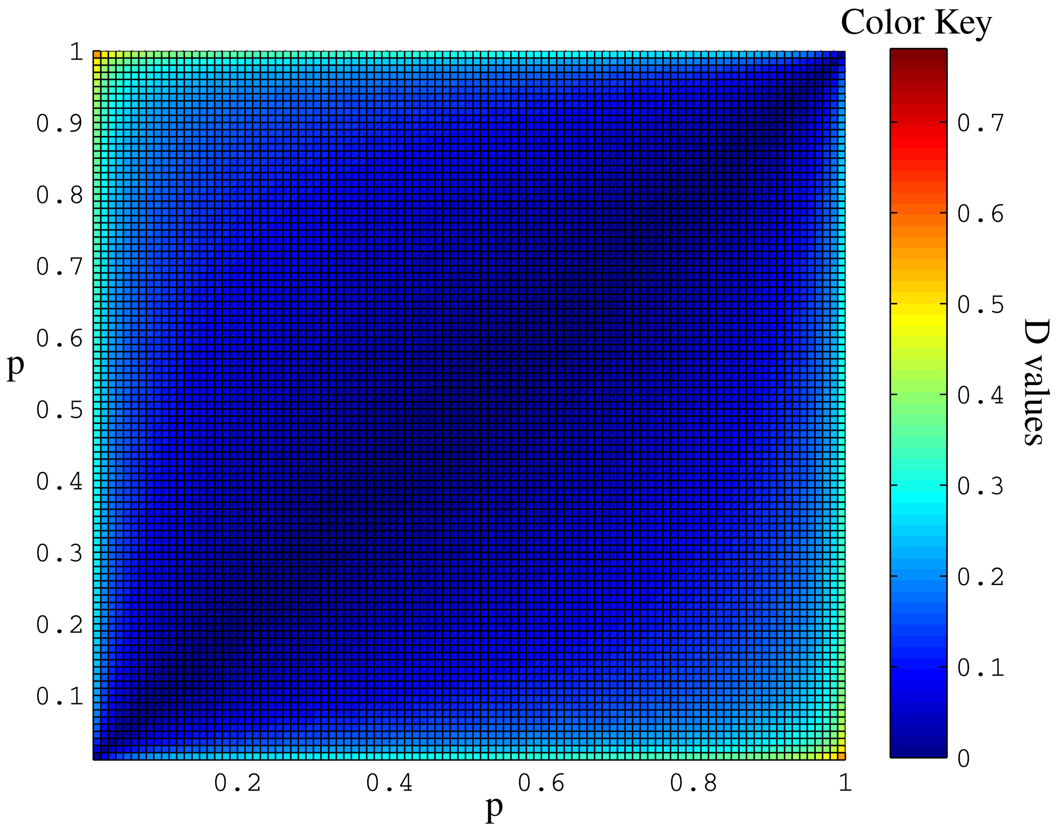

Supplementary Figure 4: **Alpha dissimilarities for the evolution Erdös-Rényi process.** Heatmap of the dissimilarity function considering weights $w_1 = w_2 = 0$, and $w_3 = 1$. The alpha centrality is unable to detect the percolation phase transition for the ER process.

The use of the alpha centrality over the graphs complements has a computational drawback. The comparison between large sparse graphs is computationally prohibitive, as their complements are dense graphs. However, as in this case, the third term do not significantly increase the value of D, it can be avoided without losing precision.

After fixing $w_3 = 0.1$, we perform a third experiment varying w_1 and w_2. The relation between w_1 and w_2 is linear, and the choice of these values depends on the importance we want to give to global and local topological features. We analyze the D values when varying w_1 and w_2, for all the cases of the classical models experiment, presented in Fig. 3 of the main text. Supplementary Fig. 5 shows the average values of 1,000 runs for each pair of networks, $D(G, G', (0.9 - w_2), w_2, 0.1)$. As is it possible to see, both terms are in the same order of magnitude, and in all cases, the relative relationship between the networks do not present strong dependency on the weights selection. The comparison of networks global (first term) and local (second term) features are highly important for having a precise general measure. We consider, for the definition of D, these features as equally important. Nevertheless, if one needs to emphasize the contribution of one of these features, the weights can be manipulated without losing the properties discussed in this manuscript.

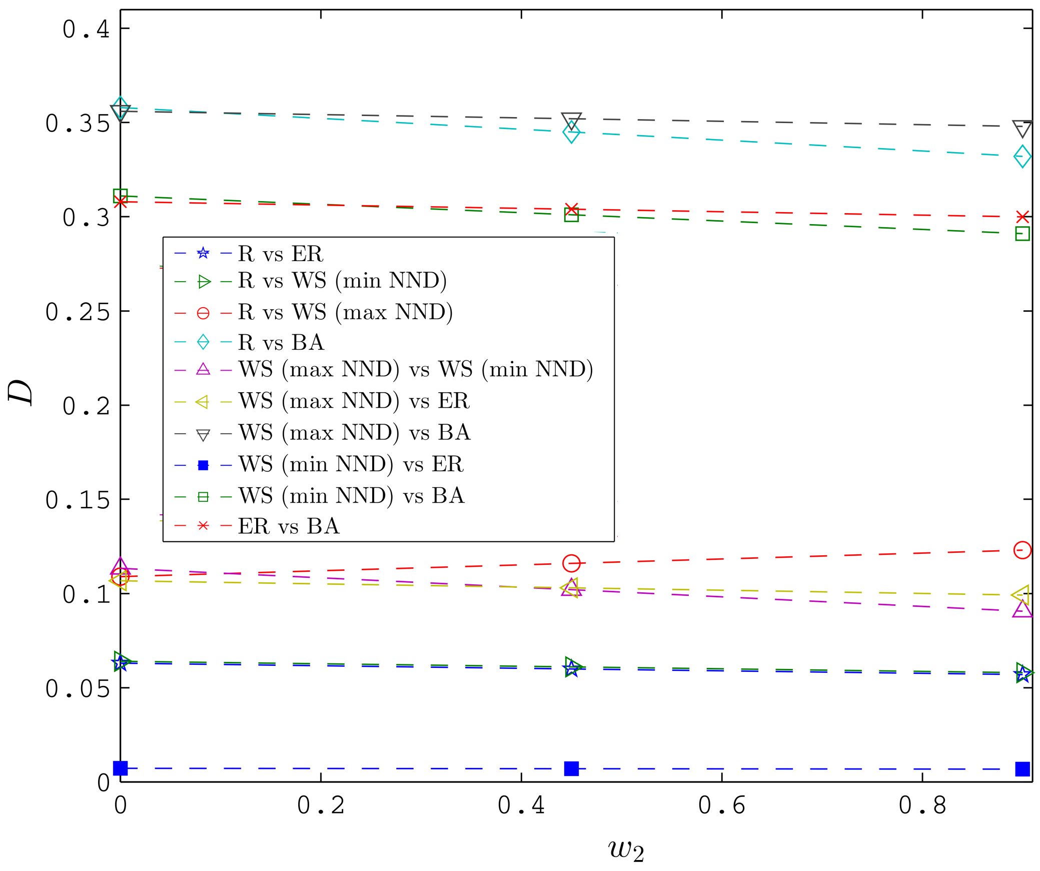

Supplementary Figure 5: **Weights analysis.** Comparisons between all the following networks: ER, Regular, BA, WS (max NND) and WS (min NND), considering $D(G, G', (0.9 - w_2), w_2, 0.1)$.

SUPPLEMENTARY NOTE 4. DISTANCE BETWEEN GRAPHS OF DIFFERENT SIZES

Given two graphs of different sizes ($N < M$), we briefly describe how to compute the dissimilarity between them.

The first term on the right-hand side of Equation (5) compares the distance distributions between them. It is easy to see that both distributions are immersed in the same probability space.

The second term on the right-hand side of Equation (5) computes the NND difference value, a size independent measure between the networks.

The third term compares alpha centrality measures. We simply add $(M - N)$ zeros to the beginning of the smaller probability distribution vector given by Equation 4. This approach is justified by the immersion of the smaller network into a larger space by adding nodes with a zero centrality value. Regarding the comparisons of full graphs (analogously empty graphs) the third term is the only capable to capture differences of them. For example, two full graphs of sizes two (G_2) and three (G_3) the PDF associated with the alpha-centrality given by Equation 4 are given by the vectors:

$$\mathbf{P}_\alpha(G_2) = (1/2, 1/2, 0) \qquad \text{and} \qquad \mathbf{P}_\alpha(G_2^c) = (0, 0, 1)$$

$$\mathbf{P}_\alpha(G_3) = (1/3, 1/3, 1/3, 0) \qquad \text{and} \qquad \mathbf{P}_\alpha(G_3^c) = (0, 0, 0, 1).$$

We transform $\mathbf{P}_\alpha(G_2)$ and $\mathbf{P}_\alpha(G_2^c)$ by adding a zero value at the beginning of the probability distribution vectors:

$$\mathbf{P}_\alpha(G_2) = (0, 1/2, 1/2, 0) \qquad \text{and} \qquad \mathbf{P}_\alpha(G_2^c) = (0, 0, 0, 1)$$

and, in this way, the computation of the last term of the right-hand side of Equation (5) can be performed.

We include an experiment starting with a 10 nodes full graph (G_{10}), then, we generate full graphs increasing the number of nodes in 20%. Finally, we compute D against G_{10} and against the complete graph considering 1/3 of the nodes $G_{N/3}$. The metric is able to distinguish all pairs of graphs, increasing the distance to G_{10} as the graphs grow, and presenting a constant distance against ($G_{N/3}$), see Supplementary Fig. 6.

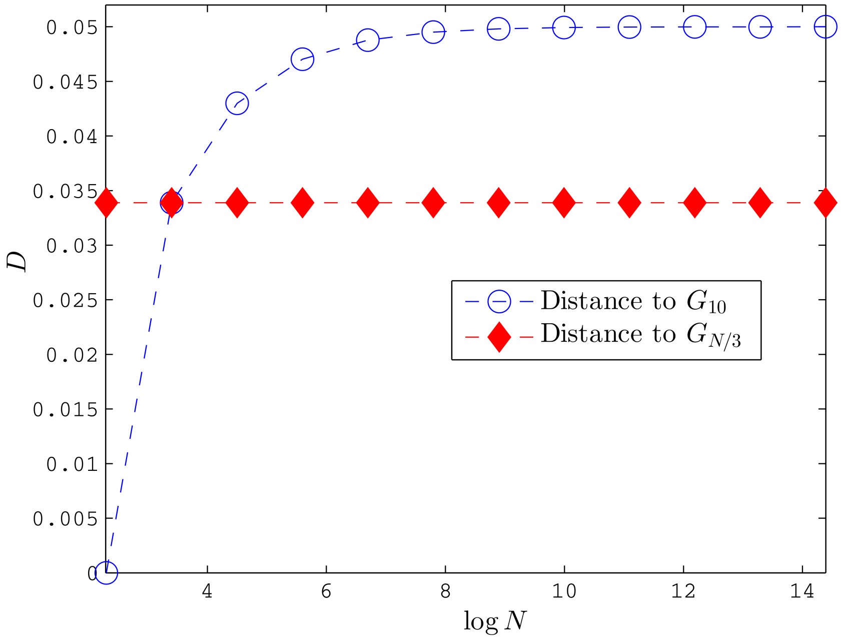

Supplementary Figure 6: **Full graphs analysis.** In this figure we compute the distance between each complete graph against a complete graph with 10 nodes, and against a complete graph with a third of the nodes considered.

SUPPLEMENTARY NOTE 5. COMPARISON OF ALGORITHMS FOR ISOMORPHISM AND DISSIMILARITIES.

Supplementary Tables 1 and 2 depict the main algorithms and methods to detect graph isomorphism. In these tables we briefly describe the methods and their major drawbacks. It is important do remark that [3] gives several classical graph metrics but, all of them are considered in Supplementary Tables 1 and 2. Some measures are equivalent to the Hamming distance in the sense that they consider the identification of the nodes. In particular, the Weight Distance, counts the number of edges, not common in both graphs with a normalization factor that results in a measure between 0 and 1, a normalized version of the Hamming Distance. Other measures, contained in this reference, are based on feature vector extraction and are included in the two first citations of Supplementary Table 2.

In order to generate a dataset for network comparisons we use nauty and Traces, programs for computing automorphism groups of graphs and digraphs [4]. In particular, there is a suite of programs called gtools included in the package that can, via geng, generate non-isomorphic graphs with a desired characteristics.

Supplementary Table 1: **Exact graph methods comparisons**

**Type**	**Description/Drawbacks**	Size Independent	Metric	Polynomial time	References
Isomorphism problem	Recently proved to be computed in quasi-polynomial time ($O(exp((\log N)^{O(1)}))$. The best previous bound was $exp(O(\sqrt{N \log N}))$. Binary answer.	Yes	No	No	[5]
Distance metrics via maximum common subgraph	Associates a cost function after finding the maximum common subgraph between two graphs. Time complexity: $O((N+1)!)$.	Yes	Yes	No	[6–8]
Graph Edit Distance	Do not capture relevant topological differences. Graph edit distance computation is equivalent to solving the maximum common subgraph problem when used with unlabeled nodes.	Yes	Yes	No	[6, 9]
Hamming distance	The time complexity is $O(N^2)$ for labeled nodes. For unlabeled nodes the computation is equivalent to solving the maximum common subgraph problem.	No	Yes	No	[10]

Supplementary Table 2: **Heuristic comparisons.** Column **SI** indicates if the method is size independent, **Metric** if the dissimilarity value has metric properties, **PT** if the algorithm runs in polynomial time, N_9 and N_{20} indicates the number of non-distinguishable graphs over the exhaustively generated set of non-isomorphic graphs of size 9 and 20, as in [11–13] [a], the symbol NT indicates that the method was not tested in this condition.

**Type**	**Description/Drawbacks**	**SI**	**Metric**	**PT**	N_9	N_{20}	**References**
Eigenvalues (spectral measures)	A network is represented as a vector containing the eigenvalues of its adjacency or Laplacian matrix. The main drawback is the existence of cospectral graphs having quite different topological structure.	No	Yes	Yes	$51,039$	NT	[14–16]
LBD, d-RW-Dist, NetSimile, NetSimile-SVM, NetSimile-Dist	It is assigned for each network a feature vector of characteristics. The runtime complexity for generating vectors is linear on the number of edges but, the algorithm's time complexity depends on which features are considered when creating the feature vector.	Yes	Yes	Feature dependent	NT	NT	[17]
Graph Kernels	Given by a kernel function that computes an inner product on graphs without having to do feature extraction. The information lacks in expressiveness when trivial features are considered. The well known random walk kernel is not precise and isomorphic networks may be considered non-isomorphic.	Yes	Yes	Yes	NT	NT	[18–22]
Balaban Index J	The Balaban index J is a graph index defined for a given distance matrix. The main drawback consists in finding 9,476,268 non-distinguishable networks of size 10.	No	No	Yes	$165,109$	NT	[12, 23]
Heuristic approach combining graph's invariants	Use of discriminating numbers to combine several different graph measures that are computable in polynomial time. The best estimate gives 993 non-distinguishable networks of size 9 (SI_{1p}, SI_{1s} and SI_{1v}).	No	No	Yes	993	NT	[11]
Hosoya-Spectral indices and the Hosoya information content	The method can distinguish all trees of size 18 but, fails in distinguishing networks of size 9. The best estimate (HS_2) gives 18 non-distinguishable networks of size 9.	No	No	Yes	18	NT	[13]
**Dissimilarity** D	**Highly discriminating but, cannot guarantee the isomorphism condition. Can distinguish all non-isomorphic graphs of size 6, 7, 8, and 9. For graphs with 20 nodes, can distinguish all tested networks in the worst cases: k-regular connected graphs with degree varying from 2 to 11. Can also distinguish all non-isomorphic trees with 20 and 21 nodes.**	**Yes**	**Yes**	**Yes**	0	**0**	**Proposed is this work**

[a] Not all works have performed this massive experiment

Supplementary Table 3: **Dissimilarity values between classical models.** Dissimilarity average values and confidence intervals computed for each pair of networks shown in Fig. 3. The table is organized by increasing averaged values. The confidence intervals are computed at 95% over $1,000$ independent experiments. Gray cells within the same column indicate no statistical significance.

R ($k = 4$)	WSMAX (max NND)	WSMIN (min NND)	ER ($p = 4/19$)	BA ($m = 2$)
ER 0.060 (0.0551,0.0651)	WSMIN 0.102 (0.0954,0.1087)	ER 0.007 (0.0062,0.0078)	WSMIN 0.007 (0.0062,0.0078)	WSMIN 0.301 (0.2952,0.3069)
WSMIN 0.061 (0.0564,0.0652)	ER 0.103 (0.0957,0.1103)	R 0.060 (0.0564,0.0652)	R 0.061 (0.0551,0.0651)	ER 0.304 (0.2972,0.3108)
WSMAX 0.116 (0.1131,0.1186)	R 0.116 (0.1131,0.1186)	WSMAX 0.102 (0.0954,0.1087)	WSMAX 0.103 (0.0957,0.1103)	R 0.345 (0.3422,0.3478)
BA 0.345 (0.3422,0.3478)	BA 0.352 (0.3462,0.3578)	BA 0.301 (0.2952,0.3069)	BA 0.304 (0.2972,0.3108)	WSMAX 0.352 (0.3462,0.3578)

SUPPLEMENTARY NOTE 6. PERCOLATION

We perform the MC simulation for the Power Grid network. Results show that the susceptibility function of the Power Grid network after 10,000 Monte Carlo simulations, contains a double phase transition characterized in lower(s) thresholds, that does not coincide with the largest percolation strength, see Supplementary Figure 7.

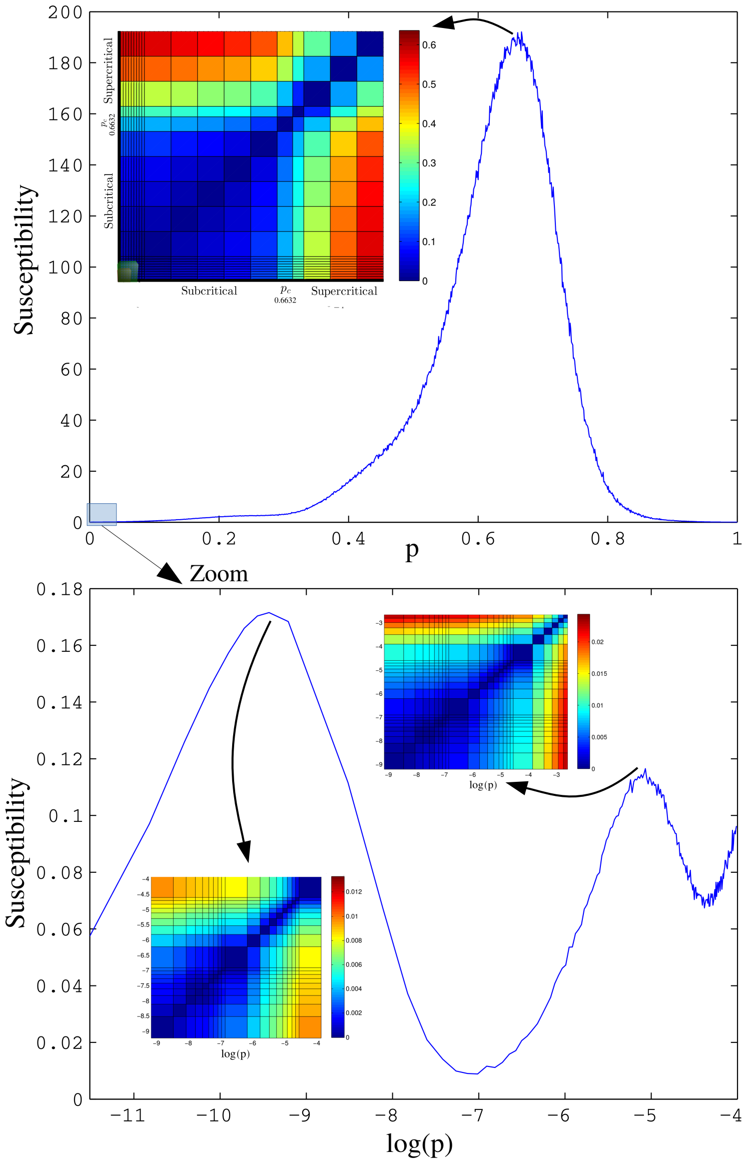

Supplementary Figure 7: **Susceptibility analysis on the Power grid network.** Susceptibility function of the Power Grid network after 10,000 Monte Carlo simulations. It contains a double phase transition characterized by lower(s) thresholds that does not coincide with the largest percolation strength. $p_c^{small(1)} 8 \times 10^{-5}$ and $p_c^{small(1)} 6 \times 10^{-3}$.

The algorithm begins with two probabilities, β and α, respectively on the supercritical and subcritical phases, and $\epsilon > 0$, a precision value. For each bond probability (α and β), we generate s networks by the random removal of an edge, independently, with probability equals one minus the bond probability. We then suppose that a network representative of the subcritical (supercritical) regime α (β), G_α (G_β) possesses distance distribution, NND, alpha centrality and alpha centrality of its complement, given by the averaged values obtained by the s generated networks.

We then, compute the mean value of these probabilities $p_m = \frac{\beta - \alpha}{2}$, and repeat the previous method to get a graph, G_m, representative for the probability p_m. If $D(G_m, G_\alpha) > D(G_m, G_\beta)$ then $\beta = p_m$ else $\alpha = p_m$, when the distance

between β and α reaches the precision value (ϵ), the algorithm stops returning $\hat{p}_c = \frac{(\beta-\alpha)}{2}$.

It is important to observe that after I iteractions, the size of the resulting interval of search is $(\beta - \alpha)/2^I$ and thus, for example, for a precision value of $\epsilon = 0.01$, $\alpha = 0$ and $\beta = 1$ we just need to perform 7 iteractions.

The time complexity of the algorithm is $\mathcal{O}(sN^3)$.

The values obtained in Table III in the main text were obtained considering $\alpha = 1/N$, $\beta = 1$ and $s = 100$. When compared with the MC simulation achieved, in average, 7 times faster. For the Power Grid network this algorithm takes 5,600 seconds to achieve the value in Table III in the main text while the MC simulation takes, after 10,000 simulations for each p value approximately 35,000 seconds.

SUPPLEMENTARY NOTE 7. EQUIVALENCE OF NULL MODELS: DK1, MS AND MR

The experiment consists in the following. We ran MR, MS increasing the number of rewiring per edge, and the dk model (k=1), for real networks. The experiment in the main text, focus on the different *dk* models. In fact, the use of the dissimilarity is a good method to detect the number of rewiring operations needed in the MS model. For example, in the case of the Power Grid network Supplementary Fig. 8, MS requires 9 times the number of edges rewiring operations to reach the same values of *dk* ($k = 1$), and MR null models. Similar results are found when testing other real networks.

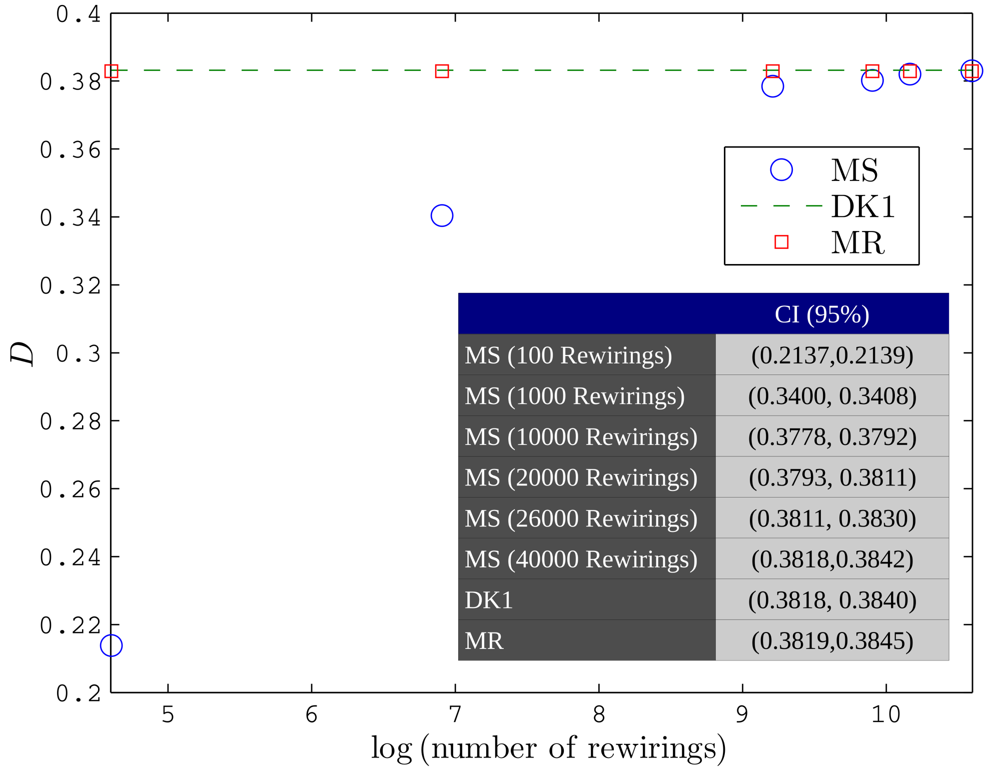

Supplementary Figure 8: **Maslov-Sneppen rewiring analysis.** Considering the Power grid network, the figure shows the distance of Molloy-Reed, *dk* and Maslov-Sneppen null models to the Power Grid network. Blue circles denote the distance of the MS model for different number rewiring operations.

Supplementary Table 4: **Dissimilarities between real networks and models.** Dissimilarity between real-world networks and four null models. From left to right we report the 95% CI interval results after 30 independent runs, DS, Degree Sequence (MS, MR, and k=1.0) generates equivalent results, the last three columns are obtained using the dk-model for k values $(2.0, 2.1, 2.5)$. We use the Shapiro-Wilk test of normality in this data.

	dk1	dk2.0	dk2.1	dk2.5
Astroph	(0.2186,0.2198)	(0.0825,0.0839)	(0.0065,0.0099)	(0.0063,0.0097)
Caida	(0.0865,0.0869)	(0.0815,0.0820)	(0.0745,0.0758)	(0.0471,0.0496)
Hypertext	(0.0092,0.0163)	(0.0013,0.0022)	(0.0016,0.0024)	(0.0017,0.0025)
Infectious	(0.2160,0.2171)	(0.1967,0.1973)	(0.1520,0.1553)	(0.1512,0.1539)
UC Irvine	(0.0333,0.0360)	(0.0234,0.0257)	(0.0227,0.0240)	(0.0217,0.0234)
Jazz	(0.1086,0.1118)	(0.0711,0.0724)	(0.0179,0.0227)	(0.0202,0.0249)
Karate	(0.1024,0.1128)	(0.1052,0.1146)	(0.0685,0.0771)	(0.0601,0.0683)
Lexical	(0.0364,0.0445)	(0.0153,0.0186)	(0.0135,0.0167)	(0.0133,0.0168)
Petster (C)	(0.2679,0.2710)	(0.1064,0.1094)	(0.0467,0.0528)	(0.0629,0.0681)
Petster	(0.1541,0.1592)	(0.0930,0.0943)	(0.0889,0.0904)	(0.0874,0.0891)
PGP	(0.3002,0.3022)	(0.2323,0.2346)	(0.2273,0.2305)	(0.1728,0.1770)
Power Grid	(0.3817,0.3840)	(0.3860,0.3883)	(0.3888,0.3910)	(0.3475,0.3498)
U. Rovirat	(0.0895,0.0914)	(0.0638,0.0655)	(0.0738,0.0786)	(0.0379,0.0409)
Yeast	(0.1040,0.1076)	(0.0799,0.0827)	(0.0662,0.0704)	(0.0468,0.0507)
Euroroad	(0.2956,0.3011)	(0.2604,0.2674)	(0.2593,0.2646)	(0.2499,0.2563)
Contiguous	(0.2238,0.2321)	(0.2031,0.2092)	(0.1235,0.1521)	(0.1152,0.1314)

SUPPLEMENTARY NOTE 8. DESCRIPTION OF THE REAL DATASETS

All networks here presented are freely available at The Koblenz Network Collection [24]. The descriptions here presented can also be found at the Koblenz website (http://konect.uni-koblenz.de/).

CAIDA: This is the undirected network of autonomous systems of the Internet connected with each other from the CAIDA project, collected in 2007. Nodes are autonomous systems (AS), and edges denote communication [24, 25]. It contains 26,475 nodes, 53,381 edges, transitivity 0.007318732, average path length 3.875647 and diameter 17.

PGP: This is the interaction network of users of the Pretty Good Privacy (PGP) algorithm. The network contains only the giant connected component of the network [24, 26]. It contains 10,680 nodes, 24,316 edges, transitivity 0.378024687, average path length 7.48554 and diameter 24.

ROVIRA: This is the email communication network at the University Rovira i Virgili in Tarragona in the south of Catalonia in Spain. Nodes are users and each edge represents that at least one email was sent [24, 27]. It contains 1,113 nodes, 5,451 edges, transitivity 0.16625006, average path length 3.606032 and diameter 8.

IRVINE: This undirected network contains sent messages between the users of an online community of students from the University of California, Irvine. A node represents a user and each edge represents that at least one email was sent [24, 28]. It contains 1,899 nodes, 59,835 edges, transitivity 0.0568303, average path length 3.0551636 and diameter 8.

JAZZ: This is the collaboration network between Jazz musicians. Each node is a Jazz musician and an edge denotes that two musicians have played together in a band [24, 29]. It contains 198 nodes, 2742 edges, transitivity 0.5202593, average path length 2.2350408 and diameter 6.

KARATE: This is the well-known Zachary karate club network. The data was collected from the members of a university karate club by Wayne Zachary in 1977. Each node represents a member of the club, and each edge represents a tie between two members of the club [24, 30]. It contains 34 nodes, 78 edges, transitivity 0.2556818, average path length 2.4081996 and diameter 5.

HYPERTEXT: This is the network of face-to-face contacts of the attendees of the ACM Hypertext 2009 conference. In the network, a node represents a conference visitor, and an edge represents a face-to-face contact that was active for at least 20 seconds. The network is considered unweighted [24, 31]. It contains 113 nodes, 2,196 edges, transitivity 0.4952046, average path length 1.6562895 and diameter 3.

INFECTIOUS: This network describes the face-to-face behavior of people during the exhibition INFECTIOUS: STAY AWAY in 2009 at the Science Gallery in Dublin. Nodes represent exhibition visitors; edges represent face-to-face contacts that were active for at least 20 seconds. The network contains the data from the day with the most interactions and is considered undirected and unweighted [24, 31]. It contains 410 nodes, 2,765 edges, transitivity 0.4356933, average path length 3.6308546 and diameter 9.

CONTIGUOUS: These are the 48 contiguous states and the District of Columbia of the United States of America (the USA). They include all states except the states of Alaska and Hawaii, which are not connected by land with the other states, and include the District of Columbia (DC). An edge denotes that two states share a border. The US states in the configuration given by this dataset exist since February 14, 1912, when Arizona was admitted as the 48th state, and is current as of 2014. The states of Alaska and Hawaii were admitted as the 49th and 50th states in 1959, but are not contiguous with the other states, and are not reflected in this dataset [24, 32]. It contains 49 nodes, 107 edges, transitivity 0.4061758, average path length 4.1632653 and diameter 11.

EUROROAD: This is the international E-road network, a road network located mostly in Europe. The network is undirected; nodes represent cities and an edge between two nodes denotes that they are connected by an E-road [24, 33]. It contains 1,174 nodes, 1,417 edges, transitivity 0.03388634, average path length 18.37129 and diameter 62.

POWER GRID: This undirected network contains information about the power grid of the Western States of the United States of America. An edge represents a power supply line. A node is either a generator, a transformator or a substation [24, 34]. It contains 4,941 nodes, 6,594 edges, transitivity 0.1031532, average path length 18.9891854 and diameter 46.

LEXICAL: This is the undirected network of common noun and adjective adjacencies for the novel "David Copperfield" by English 19th century writer Charles Dickens. A node represents either a noun or an adjective. An edge connects two words that occur in adjacent positions. The network is not bipartite, i.e., there are edges connecting adjectives with adjectives, nouns with nouns and adjectives with nouns [24, 35]. It contains 112 nodes, 425 edges, transitivity 0.156935, average path length 2.535553 and diameter 5.

YEAST: This undirected network contains protein interactions contained in yeast. A node represents a protein and an edge represents a metabolic interaction between two proteins [24, 36]. It contains 1,846 nodes, 2,203 edges, transitivity 0.0550095, average path length 6.81030 and diameter 19.

PETSTER (C): This Network contains friendships and family links between users of the website hamsterster.com [24]. It contains 2, 426 nodes, 16, 631 edges, transitivity 0.2314317, average path length 3.588250 and diameter 10.

PETSTER: This Network contains the giant connected component of the friendships and family links between users of the website hamsterster.com [24]. It contains 1, 858 nodes, 12, 534 edges, transitivity 0.09040339, average path length 3.452511 and diameter 14.

ASTROPH: This is the collaboration graph of authors of scientific papers from the arXiv's Astrophysics (astro-ph) section. An edge between two authors represents a common publication [24, 25]. It contains 18, 771 nodes, 198, 050 edges, transitivity 0.3180016, average path length 4.193988 and diameter 14.

SUPPLEMENTARY NOTE 9. THE POWER GRID AND EUROROAD NETWORKS: THE FBM EXPERIMENT

We generate 10 fractional Brownian motion (fBm) time series and construct the corresponding networks via the HVG algorithm, for different Hurst exponent (H). fBm networks posses the same size of the Power Grid and Euroroad networks. We then compute D values between the generated networks, and the Power Grid and Euroroad networks. Supplementary Table 5 depicts the averaged results.

Supplementary Table 5: **Power Grid and Euroroad networks.** Averaged dissimilarity values between fBm networks and infrastructure networks, considering different Hurst exponent values.

	$h = 0.10$	$h = 0.11$	$h = 0.12$	$h = 0.13$	$h = 0.14$
Power Grid	0.0859	0.0811	0.0902	0.0851	0.0785
Euroroad	0.2631	0.2343	0.2067	0.2301	0.2568

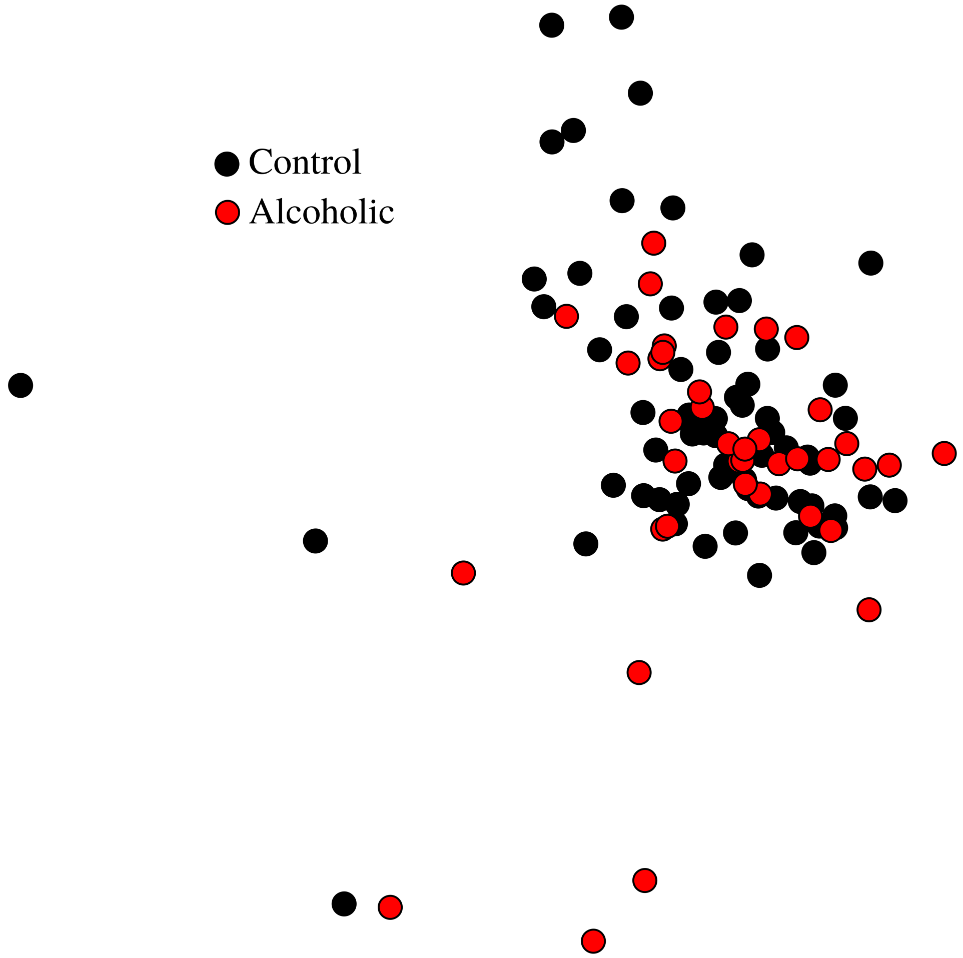

Supplementary Figure 9: **Brain application results with Hamming distance.** Results considering the methodology described in the main text, but using the Hamming distance instead of the D-measure.

SUPPLEMENTARY REFERENCES

[1] Costa, L. da F., Rodrigues, F. A., Travieso,G. & Villas Boas, P. R. Characterization of complex networks: A survey of measurements. *Adv. Phys.* **56**, 167–242 (2007).

[2] Lin, J. Divergence measures based on the Shannon entropy. *IEEE T. Inform. Theory* **37**, 145–151 (1991).

[3] Pincombe, B. Anomaly Detection in Time Series of Graphs using ARMA Processes. *ASOR Bulletin* **24**, 2–10 (2005).

[4] McKay, B. D. & Piperno, A. Practical Graph Isomorphism, II, *J. Symb. Comput.* **60**, 94–112 (2014).

[5] Babai, L. Graph Isomorphism in Quasipolynomial Time. *ArXiv* 1512.03547v2 (2016).

[6] Bunke, H. & Shearer, K. A graph distance metric based on the maximal common subgraph. *Pattern Recogn. Lett.* **19**, 255–259 (1998).

[7] Fernandez, M. L. & Valiente, G. A graph distance metric combining maximum common subgraph and minimum common supergraph. *Pattern Recogn. Lett.* **22**, 753–758 (2001).

[8] Conte, D., Pasquale, D. & Vento, M. Challenging Complexity of Maximum Common Subgraph Detection Algorithms: A Performance Analysis of Three Algorithms on a Wide Database of Graphs. *J. Graph Algorithms Appl.* **1**, 99–143 (2007).

[9] Sanfeliu, A. & Fu, K. S. A distance measure between attributed relational graphs for pattern recognition. *IEEE T. Syst. Man Cyb.* **13**, 353–363 (1983).

[10] Wilson, R. C. & Hancock, E. R. Structural matching by discrete relaxation. *IEEE T. Pattern Anal.* **6**, 634–648 (1997).

[11] Dehmer, M., Grabner, M., Mowshowitz, A. & Emmert-Streib, F. An efficient heuristic approach to detecting graph isomorphism based on combinations of highly discriminating invariants. *Adv. Comput. Math.* **2**, 311–325 (2013).
[12] M. Dehmer & Y. Shi. The Uniqueness of DMAX - Matrix Graph Invariants. *Plos One* **9**, e83868 (2014).
[13] Dehmer, M., Mowshowitz, A. & Shi, Y. Structural Differentiation of Graphs Using Hosoya-Based Indices. *PLoS One* **7**, e102459 (2014).
[14] Macindoe, O & Richards, W. Graph Comparison Using Fine Structure Analysis. *Social Computing (SocialCom), IEEE Second International Conference on*, 193–200 (2010).
[15] Mieghem, P. V. Graph Spectra for Complex Networks. New York, USA. Cambridge University Press (2011).
[16] De Domenico, M., Nicosia, V., Arenas, A. & Latora, V. Structural reducibility of multilayer networks. *Nat. Commun.* **6**, 6864 (2015).
[17] Berlingerio, M., Koutra, D., Eliassi-Rad, T. & Faloutsos, C. NetSimile: A Scalable Approach to Size-Independent Network Similarity. *WIN 2012, Workshop on Information in Networks*, New York, NY, USA (2012).
[18] Vishwanathan, S. V. N., Schraudolph, N. N., Kondor, R. & Borgwardt, K. M. Graph Kernels. *J. Mach. Learn. Res.* **11**, 1201–1242 (2010).
[19] Yanardag, P. & Vishwanathan, S.V.N. Deep Graph Kernels. *Proceedings of the 21th ACM SIGKDD International Conference on Knowledge Discovery and Data Mining. KDD '15*. New York, NY, USA (2015).
[20] Gärtner, T., Flach, P. & Wrobel, S. On graph kernels: Hardness results and efficient alternatives. *Proceedings of the 16th Annual Conference on Learning Theory* 129–143 (2003).
[21] Wu, F-X, Wu, L., Wang, J., Liu, J. & Chen, L. Transittability of complex networks and its applications to regulatory biomolecular networks. *Sci. Rep.* **4**, 4819 (2014).
[22] Hagberg, A. & Lemons, N. Fast Generation of Sparse Random Kernel Graphs. *PLoS One* **9**, 1–12 (2015).
[23] Balaban, A. T. Highly Discriminating Distance-Based Topological Index. *Chem. Phys. Lett.* **89**, 399–404 (1982).
[24] Kunegis, J. The Koblenz Network Collection. *Proc. Int. Web Observatory Workshop*, 1343–1350 (2013).
[25] Leskovec, J., Kleinberg, J. & Faloutsos, C. Graph Evolution: Densification and Shrinking Diameters. *ACM Trans. Knowledge Discovery from Data* **1**, 1–40 (2007).
[26] Boguñá, M., Pastor-Satorras, R., Díaz-Guilera, A. & Arenas, A. Models of Social Networks based on Social Distance Attachment. *Phys. Rev. E* **70**, 056122 (2004).
[27] Guimerà, R., Danon, L., DÍaz-Guilera, A., Giralt, F. & A. Arenas. Self-similar community structure in a network of human interactions. *Phys. Rev. E.* **6**, 065103 (2003).
[28] Opsahl, T. & Panzarasa, P. Clustering in weighted networks. *Social Networks* **31**, 155–163 (2009).
[29] Gleiser, P. M. & Danon, L. Community structure in jazz. *Adv. Complex Syst.* **6**, 565–573 (2003).
[30] Zachary, W. An information flow model for conflict and fission in small groups. *J. Anthropol. Res.* **33**, 452–73 (1977).
[31] Isella, L., Stehlé, J., Barrat, A., Cattuto, C., Pinton, J-F & Van den Broeck, W. What's in a crowd? analysis of face-to-face behavioral networks. *J. Theor. Biol.* **271**, 166–180 (2011).
[32] Knuth, D. E. The Art of Computer Programming. Redwood City, CA, USA. Addison-Wesley (2008).
[33] Subelj, L. & Bajec, M. Robust network community detection using balanced propagation. *Eur. Phys. J. B* **81**, 353–362 (2011).
[34] Watts, D. J. & Strogatz, S. H. Collective dynamics of small-world networks. *Nature* **393**, 440–442 (1998).
[35] Newman, M. E. J. Finding community structure in networks using the eigenvectors of matrices. *Phys. Rev. E.* **74**, 036104 (2006).
[36] Han, J. D., Dupuy, D., Bertin, N., Cusick, M. E. & Vidal, M. Effect of Sampling on Topology Predictions of Protein-protein Interaction Networks. *Nat. Biotechnol.* **23**, 839–844 (2005).
